# Household members’ positive personality traits and age stereotypes do not predict perceived expectations for active aging

**DOI:** 10.1007/s10433-025-00850-4

**Published:** 2025-03-19

**Authors:** Sonja Radoš, Maria K. Pavlova, Klaus Rothermund, Rainer K. Silbereisen

**Affiliations:** 1 Faculty of Educational and Social Sciences, Institute of Gerontology, University of Vechta, Driverstraße 23, 49377 Vechta, Germany; 2 Department of Psychology, Friedrich Schiller University of Jena, Jena, Germany

**Keywords:** Active aging, Conscientiousness, Dispositional optimism, Dyadic and interpersonal effects, Life goals, Prescriptive age stereotypes

## Abstract

The household represents a proximal social context whose members can convey various expectations to each other, including expectations for active aging. We used a nationally representative sample (*N* = 2007, aged 16–94 years) to investigate the household predictors of perceived expectations for active aging (PEAA, i.e., “activation demands” targeting individuals as older adults) in three domains: physical health, mental health, and social engagement. We considered household members’ dispositional optimism, conscientiousness, age stereotypes, and the life goal of civic engagement. A set of preregistered multiple regression analyses indicated that, irrespective of age, household members’ life goal of civic engagement had a positive effect on individual PEAA in the social engagement domain, which disappeared upon controlling for the respective individual life goal. In middle-aged and older adults, household members’ conscientiousness unexpectedly had a significantly negative effect on individual PEAA in the physical health domain. Neither household members’ dispositional optimism nor their domain-specific age stereotypes had significant associations with individual PEAA. Our findings suggest that household members’ mindsets and attitudes play a limited role in predicting PEAA of individuals from the same household.

**Supplementary Information:**

The online version contains supplementary material available at 10.1007/s10433-025-00850-4.

## Introduction

In recent decades, older adults face increasing social expectations to lead a healthy and active lifestyle (Pavlova and Silbereisen 2012; Wirth et al. 2023). Such expectations may partly result from the efforts of policy makers to promote active aging (Lessenich 2015; World Health Organization 2002). Nevertheless, proximal social environments, such as one’s household, may be the primary contexts in which individuals are confronted with novel expectations for active aging—or with more traditional expectations for disengagement in old age (de Paula Couto et al. 2022; Foster and Walker 2015; North and Fiske 2013; Pinquart and Silbereisen 2004). To function as a behavioral norm, the corresponding expectations first need to be perceived by the target individuals. Perceived expectations for active aging (PEAA) refer to “activation demands” that individuals perceive to target themselves as older adults (rather than older adults in general) in their everyday lives. For example, aging individuals may perceive that their friends or relatives expect them to actively maintain their health or to contribute to the public good in some way because of their (old) age (Pavlova and Silbereisen 2012). Conceptually, PEAA refer to the proximal contexts of aging as they represent perceived environment (Wahl and Gerstorf 2018).

Similar to views on aging in general (Kornadt and Rothermund 2011), PEAA were shown to be domain specific: Their average levels were higher in the domains of physical and mental health than in the social engagement domain (Pavlova et al. 2023), and partly domain-specific associations with various predictors emerged (Pavlova et al. 2023; Radoš et al. 2024a). Domain specificity illustrates the principles of contextual embeddedness and multidimensionality of the opportunities and constraints throughout adult development and aging (Baltes 1987; Wahl and Gerstorf 2018). Moreover, alignment with one’s self-views, concerns, or life goals appeared essential to PEAA. Specifically, factors such as subjective socioeconomic status and motivation for active aging had consistent associations with PEAA (Pavlova et al. 2023; Radoš et al. 2024a, 2024b). In contrast, personal resources like socioeconomic and health factors—which can be considered as the main determinants of inequalities in active aging—explained surprisingly little variance in PEAA (Pavlova and Silbereisen 2012; Pavlova et al. 2023). At the same time, the contribution of interpersonal factors (i.e., factors characteristic of an individual’s social environment or social interactions) to PEAA remains underexplored (see Pavlova et al. 2023; Radoš et al. 2024b; Wirth et al. 2023). Focusing on the household, we conducted a preregistered study^1^ to investigate whether personality, age stereotypes, and life goals of household members contributed to individual PEAA. To use the terminology of contextual aging research (Wahl and Gerstorf 2018), we addressed the link between perceived and actual characteristics of one’s proximal social environment—that is, between PEAA and independently measured household variables.

Research on age stereotypes, both descriptive and prescriptive (i.e., beliefs about what older adults are like and about how they should behave, respectively), usually assumes that stereotypes held by others have substantial consequences for older adults (Levy 2009; North and Fiske 2013; Kornadt et al. 2020; Rothermund and de Paula Couto 2024). Indeed, self-stereotypes of older individuals are thought to originate in societal stereotypes (Kornadt et al. 2020; Levy 2009). More broadly, health and well-being in old age depend on the multidimensional, multilevel relations and interactions between individuals and their proximal and distal contexts (Wahl and Gerstorf 2018). However, thus far, only two experimental studies have addressed the effects of others’ (fictive) attitudes on prescriptive views of aging (Wirth et al. 2023; Wirth et al., 2025). Research that assessed the psychological characteristics of significant others directly has largely focused on health behaviors in older adults as outcomes (e.g., Chopik et al. 2018; Chopik and Lee 2022; Huo and Kim 2024).

The household represents a context proximal to individuals, as they directly and regularly interact with physical and social aspects of the household environment (Wahl and Gerstorf 2018). People living together may adopt or value different lifestyles depending on their motivations, personality traits, and attitudes. Hence, the psychological makeup of household members may influence their behavior and communication, which, in turn, can become a source of PEAA—or of perceived expectations for disengagement, which are not considered in the present study (cf. Wirth et al. 2023). Even though other proximal social contexts, such as the neighborhood or the workplace, may likewise signal age-related expectations, the household, most often consisting of family members, remains a central developmental context throughout adulthood and may gain even more importance in old age (Carstensen 1995; Pinquart and Silbereisen 2004). Previously, older adults cohabiting with a partner were found to report higher PEAA across domains than those without a partner did, a finding supporting the notion that partners might “nudge” each other toward more self-care and activity (Pavlova and Silbereisen 2012; Pavlova et al. 2023). In the present study, we focused on the psychological characteristics of household members known to facilitate active aging (e.g., active health maintenance) in oneself and in significant others.

To start with personality, in older couples, dispositional optimism (a tendency to hold generalized positive expectations about the future; Carver et al. 2010) had positive dyadic effects on health (Chopik et al. 2018; Kim et al. 2014) and cognitive functioning (Oh et al. 2020). Likewise, conscientiousness (being responsible and rule-abiding; McCrae and Costa 2003) had positive dyadic effects on health in older couples (Chopik and Lee 2022; Nickel et al. 2017). Both traits have well-established links with one’s own active aging (Carver et al. 2010; Pocnet et al. 2021), but no significant relationship between one’s own optimism or conscientiousness and PEAA was found (Radoš et al. 2024a). In the household, optimistic individuals—also because of their positive views on aging (Turner and Hooker 2022)—may boost others’ motivation and provide opportunities for a healthy lifestyle. In turn, conscientious household members may facilitate PEAA in a more controlling fashion, for instance, via health-related reminders and tailored suggestions (Nickel et al. 2017). Thus, we hypothesized that optimism and conscientiousness of household members would be positively related to individual PEAA in both physical and mental health domains (Hypothesis 1). We did not expect any effects on individual PEAA in the social engagement domain because the traits in question are more strongly tied to health-related behaviors and outcomes than to social relations and activities (Carver et al. 2010; Pocnet et al. 2021).

With the onset of older age, descriptive age stereotypes become self-relevant (Rothermund and Brandtstädter 2003), predicting numerous outcomes in later life through self-perceptions of aging (i.e., beliefs about and experiences of one’s own aging; Cohn-Schwartz et al. 2021; Levy 2009). Presumably, these effects reflect a process of age-stereotype internalization (Rothermund and Brandtstädter 2003). The role of one’s own age stereotypes is extensively documented (for a review, see Meisner and Levy 2016, and Tully-Wilson et al. 2021), whereas the implications of age stereotypes held by (significant) others for old-age outcomes have only recently gained attention (Kornadt et al. 2020). Healthwise, several studies demonstrated that one partner’s self-perceptions of aging are associated with the other partner’s physical and mental health, especially if such aging perceptions are shared by both partners (Cohn-Schwartz et al. 2021; Huo and Kim 2024; Luo et al. 2021; Mejía et al. 2020). However, no research has yet considered the interpersonal effects of age stereotypes or self-perceptions of aging in the domain of social engagement. Age stereotypes held by household members might influence how they treat older adults and which age-related expectations they communicate. Arguably, individuals with positive age stereotypes would support or encourage active aging in older household members, thus facilitating their PEAA, whereas those with negative age stereotypes may communicate expectations for disengagement rather than for active aging (Wirth et al. 2023). Since both age stereotypes and PEAA are domain specific (Kornadt and Rothermund 2011; Pavlova et al. 2023; Wirth et al. 2023), we expected positive and domain-specific relationships between positive household age stereotypes and individual PEAA (Hypothesis 2). As the effects of age stereotypes depend on their self-relevance (Levy 2009), we hypothesized that household age stereotypes would have an effect on PEAA only in middle-aged and older (55 +) adults, whereas the effects of other household predictors would not be moderated by the target’s age (Hypothesis 3).

Lastly, we considered domain-specific motivation of household members, exemplified by the life goal of civic engagement. Radoš et al. (2024a) showed that individuals who endorsed this life goal themselves reported higher PEAA in the social engagement domain. Similarly, household members to whom this life goal is important may act as role models for others, fostering motivation or promoting opportunities for civic engagement (Wright et al. 2023). Valuing civic engagement may become a behavioral norm within a household, whereby civic engagement may be seen as a desirable activity, granting support and approval of significant others (Warburton and Terry 2000). To our knowledge, only one study, conducted in adults, addressed the dyadic effects of life goals on outcomes other than relationship quality, whereby altruism-related life goals of one partner (e.g., to help others or socially engage) were related to better health in the other (Wright et al. 2023). In our study, we expected a domain-specific effect, hypothesizing that household members’ life goal of civic engagement would be positively related to individual PEAA in the social engagement domain (Hypothesis 4).

## Method

### Participants and procedure

We used data from the Innovation Sample of the German Socio-Economic Panel (SOEP-IS), the yearly multidisciplinary representative survey of German adults (age 16 +), which is intended for user-designed research projects (Socio-Economic Panel 2024b). The SOEP complies with ethical research standards (Socio-Economic Panel 2024a). For the survey, trained interviewers select households using a random-walk algorithm and conduct standardized computer-assisted personal interviews. Participants receive monetary incentives (Socio-Economic Panel 2024a). According to the fieldwork report (Zweck and Glemser 2018), in 2016, 3049 out of the 3550 households that were approached participated (85.9%), and 4802 of the 5376 eligible individuals participated (89.3%). The interviews lasted 92.7 min on average. The majority (71%) of households in our subsample had between two and four members. In adults aged 55 + , two-person households were the most common category (61.9%). Our items on PEAA, preparation for age-related changes, and age stereotypes (Pavlova et al. 2023) were administered in 2016 to a randomly drawn subsample of 2007 individuals aged 16–94. We used household predictor variables from the same or previous survey waves. Table 1 presents the descriptive statistics.

**Table 1:** Descriptive statistics

Variable	Summary statistics
Individual PEAA: physical health (1–7), *M* (*SD*)	5.1 (1.7)
Individual PEAA: mental health (1–7), *M* (*SD*)	5.4 (1.6)
Individual PEAA: social engagement (1–7), *M* (*SD*)	3.8 (1.8)
Age (16–94), *M* (*SD*)	52.7 (18.2)
Female, *n* (%)	1039 (51.8%)
Subjective socioeconomic status (1–10), *M* (*SD*)	6.0 (1.4)
Cohabiting with a partner, *n* (%)	1313 (65.4%)
General health (1–5), *M* (*SD*)	3.4 (0.9)
HH optimism^a^ (1–7), *M* (*SD*)	4.5 (0.8)
HH conscientiousness^a^ (1–7), *M* (*SD*)	5.9 (0.8)
HH age stereotypes: physical health (1–7), *M* (*SD*)	4.2 (1.2)
HH age stereotypes: mental health (1–7), *M* (*SD*)	4.5 (1.1)
HH age stereotypes: social engagement (1–7), *M* (*SD*)	4.5 (1.2)
HH life goal: civic engagement (1–4), *M* (*SD*)	2.2 (0.8)

*N* = 2007. PEAA = perceived expectations for active aging. HH = household. Among control variables, only subjective socioeconomic status was administered to an independently selected random subsample of participants in a previous wave. Thus, only 909 participants from our subsample had valid values on this measure. Household measures had up to 35% of missing values. For each participant, household measures represented a mean of scores of their household members, which excluded target individuals’ own scores

^a^Raw mean scores are shown

## Measures

### Individual perceived expectations for active aging (PEAA)

To assess PEAA (outcome variables) in target individuals, we employed three single items adapted from the Jena Study on Social Change and Human Development (Pavlova and Silbereisen 2012), preceded by the instruction: “In the following, we want to ask you about aging and social expectations. Please think of your everyday life. In our society, we often face certain expectations of other people. The following questions relate to these expectations”. Each item had a 7-point rating scale (1 = *does not apply at all*; 7 = *applies completely*) and referred to a specific domain: physical health (“It is expected of me to keep myself physically fit”), mental health (“It is expected of me to keep myself mentally fit”), and social engagement (“People have high expectations that I get involved in social and non-profit-making activities”). Correlational analysis suggested that these items could be used separately to measure specific PEAA domains (Pavlova et al. 2023).

### Household predictors

To obtain the household values for the predictor variables (e.g., personality characteristics or age stereotypes), we computed a mean of all household members’ individual scores excluding the target participant.^2^ Hence, one and the same participant could provide the outcome (PEAA) scores as a target individual and the predictor scores as a household member of other target individuals. We did not employ PEAA of household members as predictors. We measured optimism with two items administered in 2014: “If you think about the future: Are you...” (1 = *pessimistic*, 4 = *optimistic*; Trommsdorff 1994) and “I am optimistic about my future” (1 = *completely disagree*; 7 = *fully agree*; Diener et al. 2010), *r* = 0.48. We assessed conscientiousness with three items from the Big Five section of the SOEP (Gerlitz and Schupp 2005) administered in 2015: “I am someone who... tends to be lazy,” coded inversely, “works thoroughly,” and “does things effectively and efficiently” (1 = *does not apply at all*; 7 = *fully applies*; α = 0.60). For the life goal of civic engagement, we used a single item from a set of questions about the importance of life goals administered in 2016 (previously used by Buchinger et al. 2022): “Various things can be important for various people. Are the following things currently important for you?... be politically and/or socially involved” (1 = *quite unimportant;* 4 = *very important*). Domain-specific age stereotypes were assessed with three single items in 2016: “Older people are healthy and physically fit” for physical health, “Older people are mentally fit” for mental health, and “Older people have a lot of energy and power for meaningful activities, e.g., hobbies or voluntary activities” for social engagement (1 = *does not apply at all*; 7 = *applies completely*).

### Control variables

Using participants’ chronological age in 2016, we defined five age groups: 16–24 years (emerging adults), 25–39 years (young adults), 40–54 years (middle-aged adults), 55–69 years (young-old adults), and 70 + (middle-old and older adults). Sex was a binary variable (0 = *male*; 1 = *female*). We further controlled for the variables previously identified as PEAA correlates in the same dataset (Pavlova et al. 2023), which were partnership (1 = *cohabiting with a partner*, irrespective of marital status; 0 = *not*), subjective socioeconomic status (a mean of two items from the social ladder scale; Adler et al. 2000), and general health (one item; “How would you describe your current health?”; 1 = *bad*; 5 = *very good*).

### Analytical approach

We conducted statistical analyses in Mplus 8.5 (Muthén and Muthén 2017) and modeled optimism and conscientiousness as latent variables. To test our hypotheses, we regressed each of the three outcome variables (domain-specific individual PEAA) on the predictors entered in two blocks (only control variables; control and household variables). For planned tests (i.e., where we had hypotheses), we used a conventional alpha level of 0.05, whereas for other (post-hoc) tests, we used a 0.01 level. Following observation of a significant effect of one focal predictor in at least one domain, we compared the standardized regression coefficients between the domains using a *z*-test. To test for the moderating effects of age, we dichotomized the age variable (under 55 and 55 +) and utilized a multiple-group approach to compare the unstandardized regression coefficients statistically (Muthén and Muthén 2017). To avoid multiple significance testing, we first conducted an omnibus test of model equivalence (i.e., with the coefficients of interest constrained to be equal across age groups compared with all coefficients being free to vary). To take the nonindependence of observations within households into account, we used a robust sandwich estimator, similarly to multilevel modeling in the actor–partner interdependence framework (Cook and Kenny 2005) but without explicitly modeling the household level. We handled missing values with the full information maximum likelihood estimation, which utilizes all available information from each observation without imputing missing values (Muthén and Muthén 2017).

## Results

### Measurement models

The measurement model for the personality traits of the household members included two latent variables: conscientiousness (three indicators that represented the household members’ mean scores excluding the target participant) and optimism (two indicators that were also averaged across household members). This model had an excellent fit, χ^2^ (5, *N* = 1286) = 5.6, *p* = 0.343, CFI = 0.998, RMSEA = 0.010, SRMR = 0.016, with a correlation of 0.24 between the two latent variables (see Online Resource 1 for the correlations between all predictors and outcomes).

### Regression analyses

Table 2 shows the main findings. The effects of control variables (see Model 1) were described elsewhere (cf. Pavlova et al. 2023). In Model 2, household predictors were added. As expected, the household life goal of civic engagement had a positive and significant effect on individual PEAA in the social engagement domain (β = 0.13), significantly more positive than in the mental health domain (*p* = 0.037). In supplementary analysis (not shown in tables), this effect was no longer significant after the respective individual life goal was included into the equation, *B*(*SE*) = 0.10 (0.07), *p* = 0.138. No other household predictor had significant effects in any of the domains. Therefore, Hypothesis 4 was supported, whereas Hypotheses 1 and 2 were refuted. We went on to test Hypothesis 3 (age differences; not shown in tables). Based on the omnibus tests comparing the models with the effects of household age stereotypes on individual PEAA constrained to be equal across the age groups to the models with unconstrained effects, we found no differences in any of the domains. Specifically, our results were: for the physical health domain, χ^2^ (3, *N* = 2007) = 1.6, *ns*, for the mental health domain, χ^2^ (3, *N* = 2007) = 2.2, *ns*, and for the social engagement domain, χ^2^ (3, *N* = 2007) = 4.0, *ns.*

**Table 2:** Multiple regression analyses

	PEAA: Model 1			PEAA: Model 2		
Predictor	PH	MH	SE	PH	MH	SE
*Control variables*						
Age 17–24^a^	0.45† (0.18)	0.46* (0.15)	0.60* (0.18)	0.42† (0.17)	0.57** (0.16)	0.58* (0.19)
Age 25–39^a^	− 0.10 (0.14)	− 0.16 (0.13)	0.09 (0.14)	− 0.09 (0.14)	− 0.09 (0.13)	0.02 (0.15)
Age 40–54^a^	0.15 (0.13)	0.09 (0.12)	0.32† (0.13)	0.14 (0.13)	0.14 (0.12)	0.27 (0.14)
Age 55–69^a^	0.09 (0.13)	− 0.01 (0.12)	0.29† (0.13)	0.13 (0.12)	0.05 (0.12)	0.27† (0.13)
Female	0.10 (0.08)	0.14† (0.07)	0.10 (0.08)	0.07 (0.07)	0.14† (0.07)	0.06 (0.08)
Subjective socioeconomic status	0.10† (0.05)	0.10† (0.05)	0.10 (0.05)	0.06 (0.05)	0.09† (0.04)	0.12† (0.05)
Partnership	0.33* (0.10)	0.31** (0.09)	0.31* (0.10)	0.36** (0.10)	0.28* (0.10)	0.28† (0.11)
General health	0.18* (0.05)	0.12† (0.05)	0.08 (0.06)	0.12† (0.05)	0.10† (0.05)	0.08 (0.05)
*Household predictors*						
Optimism				**0.02** **(0.18)**	**0.13** **(0.16)**	0.11 (0.22)
Conscientiousness				**−0.05** **(0.11)**	**0.06** **(0.11)**	−0.02 (0.11)
Age stereotypes: PH				**−0.01** **(0.07)**	−0.06 (0.07)	−0.13 (0.08)
Age stereotypes: MH				−0.01 (0.07)	**0.08** **(0.07)**	0.11 (0.08)
Age stereotypes: SE				0.08 (0.06)	0.06 (0.06)	**−0.02** **(0.06)**
Life goal of civic engagement				0.15† (0.07)	0.10 (0.07)	0.30*** **(0.08)**
*R*^2^	0.025	0.033	0.031	0.032	0.042	0.049
Δ*R*^2^	–	–	–	0.006	0.008	0.016

*N* = 2007. Cells show unstandardized regression coefficients with standard errors in parentheses. PEAA = perceived expectations for active aging. PH = physical health domain. MH = mental health domain. SE = social engagement domain. The coefficients used in testing our hypotheses, regardless of their significance, are shown in bold

^a^Reference group: Age 70 +

^†^*p* < 0.10 * *p* < 0.05. ** *p* < 0.01. *** *p* < 0.001. (for planned significance tests, indicated in bold)

^†^*p* < 0.05. * *p* < 0.01. ** *p* < 0.001. (for post-hoc significance tests, including those for control variables)

Unexpectedly, age differences in the effects of other predictors were significant in the physical health domain, χ^2^ (3, *N* = 2007) = 14.2, *p* < 0.01, and the social engagement domain, χ^2^ (3, *N* = 2007) = 54.3, *p* < 0.001, but not in the mental health domain, χ^2^ (3, *N* = 2007) = 10.0, *ns*. However, the only significant difference in specific regression coefficients (according to our criteria for post-hoc tests) was that for the household conscientiousness. In particular, only in adults aged 55 + , household conscientiousness had a significantly negative effect on individual PEAA in the physical health domain, *B*(*SE*) = −0.49 (0.18), *p* = 0.008, β = −0.18. Therefore, Hypothesis 3 was not supported: We found no significant age differences in the effects of age stereotypes but observed unexpected significant age differences in the effects of household conscientiousness.

### Supplementary dyadic analyses

Following the suggestion of an anonymous reviewer, we performed dyadic analyses to check whether the mindsets and attitudes of one’s cohabiting partner or spouse, rather than those of all household members, are related to individual PEAA in middle-aged and older adults in particular (aged 40 and above; see Online Supplement 2). We applied the alpha level for post-hoc analyses here. We also controlled for household composition, which was consistently associated with PEAA across domains. Specifically, individuals living in two- or multi-person households reported significantly or marginally significantly higher PEAA across domains than those living alone, βs = 0.10–0.14. The effects of the focal predictors did not substantially deviate from those reported above. Partner life goal of civic engagement remained the only significant predictor of individual PEAA in the social engagement domain,* B*(*SE*) = 0.29 (0.09), *p* = 0.001, β = 0.13, and yielded a marginally significant effect in the physical health domain, *B*(*SE*) = 0.17 (0.08), *p* = 0.043, β = 0.08. In the social engagement domain, we noted a marginally significant positive effect of partner optimism on PEAA, *B*(*SE*) = 0.49 (0.22), *p* = 0.026, β = 0.13, and a marginally significant negative effect of partner conscientiousness on PEAA, *B*(*SE*) = −0.32 (0.15), *p* = 0.034, β = −0.11. In addition, we tested the interactions between partner age stereotypes and participants’ age (middle-aged and young-old vs. middle-old adults), to further examine the possibility of age-differentiated effects on domain-specific PEAA. No significant interaction effects emerged.

## Discussion

In this preregistered study, we examined the roles of household members’ mindsets and attitudes for individual domain-specific PEAA. We found few significant effects, which suggested the surprising conclusion that personality traits and age stereotypes of one’s household members do not play a major role in conveying expectations for active aging. However, rather than undermining the developmental relevance of proximal environments, our findings corroborate the notion that objective and perceived environments may not necessarily align (Wahl and Gerstorf 2018).

Contrary to our expectations, household members’ dispositional optimism had no significant effects on PEAA in the physical and mental health domains. This null result does not align with one of the suggested pathways behind the dyadic effects of optimism on health, namely promoting healthy behavioral norms (Oh et al. 2020). Even more unexpectedly, in middle-aged and older adults, household members’ conscientiousness was *negatively* associated with PEAA in the physical health domain. Probably the kind of social support that conscientious individuals offer to their close others may sometimes have a paradoxical effect (Rook et al. 2011). Indeed, even seemingly positive features of the proximal social context can sometimes hinder, rather than promote, health and well-being in older age (Wahl and Gerstorf 2018). For example, conscientious individuals who readily take over household duties from their older family members may signal reduced expectations of an active contribution from them.

Perhaps most surprisingly, the anticipated effects of household members’ domain-specific age stereotypes on PEAA in matching domains were observed neither in the entire sample nor in adults aged 55 + in particular. Age stereotypes of one’s cohabiting partner or spouse were likewise irrelevant to PEAA. This null finding may reflect the well-known discrepancy between individuals’ attitudes and behavior and how close others perceive them. Research on interpersonal perception has consistently shown perceptual accuracy to be frequently limited (Kenny and Acitelli 2001). In a similar vein, contextual aging research emphasizes that contextual influences are not always accurately attributed within one’s perceived environment (Wahl and Gerstorf 2018). Simply put, household members may not act in ways consistent with their age stereotypes, or when they do, middle-aged and older individuals may not perceive this behavior as communicating greater (or lower) expectations of aging actively. Studies that investigated the dyadic effects of self-perceptions of aging on health-related outcomes found more individual than partner effects, with the latter commonly occurring through the other partner’s self-perceptions of aging (Cohn-Schwartz et al. 2021; Luo et al. 2021). Therefore, it is also possible that household age stereotypes are influential only as long as they align with older adults’ own age stereotypes.

Nevertheless, as descriptive age stereotypes are known to be consequential (Levy 2009; Meisner and Levy 2016; Rothermund and Brandtstädter 2003; Tully-Wilson et al. 2021), our null results raise an important question of how exactly they become internalized. Importantly, our findings challenge the commonly assumed relevance of significant others’ age stereotypes to older adults’ views on aging (Kornadt et al. 2020; North and Fiske 2013; Rothermund and de Paula Couto 2024). Another possibility is that negative age stereotypes actually lead to *increased* expectations for active aging that aim to motivate older adults to compensate for their shortcomings (e.g., “Now that you are old, you have to be more active/live healthier/invest more into your fitness than before in order to counteract age-related losses and deficits”). Such counteractive expectations might neutralize the relationship between age stereotypes of significant others and PEAA in older adults.

We did observe the anticipated positive effect of household members’ (or a partner’s) life goal of civic engagement on PEAA in the social engagement domain. However, in supplementary analyses, we found that this household effect became nonsignificant once the respective individual life goal had been controlled for. Thus, rather than attesting to a unique and direct effect of household members’ mindsets, this finding suggested that their life goal of civic engagement was linked to individual PEAA by virtue of goal correspondence (similarly to the well-known phenomenon of assortative mating; Denzinger et al. 2018). Again, this finding underscores the importance of sharing views with significant others (cf. Cohn-Schwartz et al. 2021; Mejía et al. 2020). Finally, rather than the psychological characteristics of household members, their mere presence was consistently related to somewhat increased PEAA across domains. This finding suggests that household members may “activate” each other in some straightforward way, such as coordinating the division of household labor or engaging in joint activities, which, in turn, may facilitate PEAA.

Our study had significant strengths but also several limitations. The SOEP-IS is the first nationally representative study to have included the measures of domain-specific PEAA, for which reason our findings can be generalized to the German population. However, the correlational design of our study with PEAA being assessed at only one measurement occasion precluded us from establishing causality. We preregistered our hypotheses and the analysis plan and used strict ad-hoc and post-hoc criteria in interpreting our results. Our subsample was randomly selected from a large longitudinal SOEP-IS sample of private households, with some of the data missing because of dropout or by design. We dealt with all types of missingness by using a full-information estimator. Substantial amounts (up to 35%) of missing data on household measures due to the presence of single households and to item nonresponse in two-person households may limit the strength of our conclusions. Nevertheless, linking mindsets and attitudes of household members (i.e., exemplary contextual elements of one’s proximal environment) with target individuals’ age-related perceptions was a key strength of our study. Furthermore, our sample included emerging adults (aged 16–24) who are more likely to live together with strangers (e.g., in dormitories) rather than with their significant others. For these participants, household effects may have a different meaning. However, the supplementary dyadic analyses on middle-aged and older adults and their partners did not change our conclusions. Moreover, as the mean scores on the household predictors could conceal individual differences, we tested the models with maximum and minimum (instead of average) household values and found no differences from the main findings presented above. Finally, many constructs in the SOEP-IS were assessed with few or single items. Although suitable for our domain-specific approach to age stereotypes and PEAA, this measurement issue could result in less than optimal reliability of our measures.

## Conclusion and outlook

To conclude, although proximal social environments may encourage and model desirable behaviors in various ways (Umberson et al. 2010; Wahl and Gerstorf 2018), our study showed that there is no *direct* connection between household members’ positive age stereotypes and personality traits and individual perceptions of expectations for active aging. Future studies could examine the role of the household in promoting active (or passive) aging in more depth by assessing the quality of specific relationships (e.g., partners, children). In addition, future research may address the importance of similarity (or difference) in mindsets between older individuals and their significant others. Apart from households, researchers could look at the roles of other proximal and distal contexts likely to communicate expectations for active aging (e.g., communities, leisure associations, or healthcare settings).

## Supplementary Information

Below is the link to the electronic supplementary material.

Supplementary material1 (DOCX 26 KB)

## Data Availability

We used data from the Innovation Sample of the German Socio-Economic Panel, which included our own data module approved and administered by the SOEP. To access the data, the German data protection laws require all users to sign a data distribution contract with the DIW. Further inquiries can be directed to the DIW (https://www.diw.de/en/diw_01.c.390440.en/soep_is.html).

## Footnotes

1 The preregistration can be found under https://osf.io/24pw8. This study focused on testing hypotheses H7-H10 of this preregistration.

2 We also re-ran the models described below using the maximum and minimum values of the household instead of the household average, and these analyses yielded substantially the same pattern of results.
