# Supplementary material for: Household members’ positive personality traits and age stereotypes do not predict perceived expectations for active aging
Source: Eur J Ageing. 2025 Mar 19;22(1):11. doi: 10.1007/s10433-025-00850-4 (PMC11923321; doi:10.1007/s10433-025-00850-4)
Supplement: Supplementary file 1 — Supplementary material1 (DOCX 26 KB) [file 10433_2025_850_MOESM1_ESM.docx]

Online Resource 1. Bivariate correlations between predictors and outcomes

| Variable | 1 | 2 | 3 | 4 | 5 | 6 | 7 | 8 | 9 |
| --- | --- | --- | --- | --- | --- | --- | --- | --- | --- |
| 1. Individual PEAA: physical health | — | .73** | .40** | .04 | -.02 | .08* | .03 | .03 | .06 |
| 2. Individual PEAA: mental health |  | — | .40** | .09 | .03 | .07* | .04 | .06 | .07* |
| 3. Individual PEAA: social engagement |  |  | — | .05 | -.01 | .14** | -.02 | .01 | .00 |
| 4. HH optimism |  |  |  | — | .22 | .02 | .11 | .05 | .13* |
| 5. HH conscientiousness |  |  |  |  | — | -.02 | .07 | .05 | .09* |
| 6. HH life goal: civic engagement |  |  |  |  |  | — | .06 | .06 | .12** |
| 7. HH age stereotypes: physical health |  |  |  |  |  |  | — | .73** | .58** |
| 8. HH age stereotypes: mental health |  |  |  |  |  |  |  | — | .56** |
| 9. HH age stereotypes: social engagement |  |  |  |  |  |  |  |  | — |

*Note.* PEAA = perceived expectations for active aging. HH = household. Variables 4–9 (predictors) represent mean scores for each participants’ household members. These measures excluded individual participants’ respective scores.

* *p* < .05 ***p* < .01.

Online Resource 2. Partner effects in middle-aged and older (40+) individuals

|  | PEAA: Model 1 | | | PEAA: Model 2 | | | |
| --- | --- | --- | --- | --- | --- | --- | --- |
| Predictor | PH | MH | SE | | PH | MH | SE |
| *Control variables* |  |  |  | |  |  |  |
| Age 40-54^a^ | 0.06  (0.14) | -0.00  (0.14) | 0.18  (0.16) | | 0.10  (0.15) | -0.01  (0.14) | 0.13  (0.16) |
| Age 55-69^a^ | 0.09  (0.12) | -0.01  (0.12) | 0.23  (0.13) | | 0.11  (0.12) | 0.01  (0.12) | 0.23  (0.13) |
| Female | 0.04  (0.08) | 0.11  (0.08) | -0.06  (0.09) | | 0.01  (0.09) | 0.08  (0.08) | -0.11  (0.09) |
| Subjective socioeconomic status | 0.10†  (0.05) | 0.16*  (0.05) | 0.14†  (0.06) | | 0.07  (0.06) | 0.13†  (0.06) | 0.13†  (0.06) |
| Two-person household^b^ | 0.41*  (0.13) | 0.38*  (0.12) | 0.41*  (0.13) | | 0.44*  (0.14) | 0.41*  (0.13) | 0.39*  (0.15) |
| Multi-person household^b^ | 0.41†  (0.16) | 0.43*  (0.15) | 0.45*  (0.17) | | 0.44†  (0.18) | 0.52*  (0.17) | 0.44†  (0.19) |
| General health | 0.09  (0.05) | 0.10†  (0.05) | 0.12†  (0.06) | | 0.08  (0.06) | 0.08  (0.06) | 0.09  (0.06) |
| *Partner predictors* |  |  |  | |  |  |  |
| Optimism |  |  |  | | 0.14  (0.18) | 0.29  (0.16) | 0.49†  (0.22) |
| Conscientiousness |  |  |  | | -0.27  (0.15) | -0.11  (0.13) | -0.32†  (0.15) |
| Age stereotypes: PH |  |  |  | | 0.01  (0.08) | -0.09  (0.08) | -0.12  (0.09) |
| Age stereotypes: MH |  |  |  | | -0.07  (0.08) | 0.05  (0.08) | 0.13  (0.10) |
| Age stereotypes: SE |  |  |  | | 0.12  (0.07) | 0.12  (0.07) | -0.08  (0.08) |
| Life goal of civic engagement |  |  |  | | 0.17†  (0.08) | 0.07  (0.08) | 0.29*  (0.09) |
| *R*^2^ | .026 | .042 | .037 | | .046 | .058 | .079 |
| Δ*R*^2^ | - | - | - | | .019 | .016 | .039 |

*Note*. *N* = 1,453. Cells show unstandardized regression coefficients with standard errors in parentheses. PEAA = perceived expectations for active aging. PH = physical health domain. MH = mental health domain. SE = social engagement domain. For predictor and control variables alike, we used post-hoc significance thresholds.

^a^ Reference group: Age 70+.

^b^ Reference group: Single households.

† *p* < .05. * *p* < .01. ** *p* < .001.

**Article title**: Household members’ positive personality traits and age stereotypes do not predict perceived expectations for active aging

**Journal name**: European Journal of Ageing

**Author names and affiliation**: Sonja Radoš, M.S. (University of Vechta), Maria K. Pavlova, Ph.D. (University of Vechta), Klaus Rothermund, Ph.D. (Friedrich Schiller University of Jena), and Rainer K. Silbereisen, Ph.D. (Friedrich Schiller University of Jena)

**Corresponding author**: Sonja Radoš, [sonja.rados@uni-vechta.de](mailto:sonja.rados@uni-vechta.de)
